# Mining Actinomycetes for Novel Antibiotics in the Omics Era: Are We Ready to Exploit This New Paradigm?

**DOI:** 10.3390/antibiotics7040085

**Published:** 2018-09-25

**Authors:** Olga Genilloud

**Affiliations:** Fundación MEDINA, Avda Conocimiento 34, 18016 Granada, Spain; olga.genilloud@medinaandalucia.es

**Keywords:** actinomycetes, antibiotics, secondary metabolism, culture-based approaches, omics

## Abstract

The current spread of multi-drug resistance in a number of key pathogens and the lack of therapeutic solutions in development to address most of the emerging infections in the clinic that are difficult to treat have become major concerns. Microbial natural products represent one of the most important sources for the discovery of potential new antibiotics and actinomycetes have been one of the most relevant groups that are prolific producers of these bioactive compounds. Advances in genome sequencing and bioinformatic tools have collected a wealth of knowledge on the biosynthesis of these molecules. This has revealed the broad untapped biosynthetic diversity of actinomycetes, with large genomes and the capacity to produce more molecules than previously estimated, opening new opportunities to identify the novel classes of compounds that are awaiting to be discovered. Comparative genomics, metabolomics and proteomics and the development of new analysis and genetic engineering tools provide access to the integration of new knowledge and better understanding of the physiology of actinomycetes and their tight regulation of the production of natural products antibiotics. This new paradigm is fostering the development of new genomic-driven and culture-based strategies, which aims to deliver new chemical classes of antibiotics to be developed to the clinic and replenish the exhausted pipeline of drugs for fighting the progression of infection diseases in the near future.

## 1. Introduction

Actinomycetes are accepted as one of the most relevant bacterial groups as prolific producers of secondary metabolites (SM). For decades, they were intensively exploited by industrial discovery programs that deliveredmost of the natural products, which were subsequently used as scaffolds to derive a large number of the antibiotics that are currently in the clinic [1,2]. Despite this past success, the current spread of multi-drug resistance in a number of key pathogens (*Enterococcus faecium*, *Staphylococcus aureus*, *Klebsiella pneumoniae*, *Acinetobacter baumannii*, *Pseudomonas aeruginosa* and *Enterobacter* spp.) and the lack of therapeutic solutions in development to address most of the emerging infections in the clinic that are difficult to treat have become major health concerns and require a new and sustained research effort to respond to the need for new antibiotics [3,4,5]. The progressive abandonment of the field by large pharmaceutical companies, moving away from the high costs of development and poor incentives as part of a broken economic model, has left academic groups and small biotech companies as the only players in the discovery field. These research teams are confronted with the major challenge of identifying novel antibiotic classes that they may not have the capacity to develop alone to reach the market [6,7]. The international initiatives launched in the last few years are supporting the development of new antibiotics and are revitalizing new preclinical and clinical developments. In contrast, basic research and early discovery efforts still remain poorly addressed by these programs and the existing research gaps are not enabling the establishment of a sustainable early discovery pipeline for future new compounds from the innovative approaches that have emerged in the field in last few decades [8,9]. This limited number of clinical development programs is in direct contrast to the dynamic and prolific activity that has developed in the field of microbial natural products during the last decade [10,11]. Research in natural products has evolved to represent one of the most important sources for the discovery of potential new antibiotics, compared to the lack of success of synthetic molecules. The poor suitability of synthetic libraries that were used to select molecules from target-based in vitro screens, or to develop rational drug designs based on ligand binding devoid of suitable physiochemical properties for penetrating bacterial membranes has been extensively discussed in previous reports [12,13,14,15]. Despite the rediscovery problem of known compounds, which has been represented as one of the major burdens for the continued investment in traditional NP discovery programs in the past, the advances in genome sequencing and bioinformatic tools have permitted us to collect a wealth of knowledge on the biosynthesis of natural products, which has revealed the broad untapped biosynthetic diversity of microorganisms, especially actinomycetes [16]. These talented filamenting bacteria have been shown to encode a previously unexpected diversity of biosynthetic gene clusters, opening new opportunities to identify novel classes of compounds that are awaiting to be discovered [16,17,18]. New advances in cultivation techniques from unexploited microbial niches have provided new insights into the diversity of actinobacteria and the possibility to access a broader chemical space of bioactive compounds. Comparative genomics, metabolomics and proteomics and the development of new analysis tools are generating a wealth of integrated information, which is fostering the emergence of new strategies that are aimed at obtaining a better understanding of the physiology of actinomycetes and their production of natural products. The new methods developed to mine genomes and predict chemical structures, to activate silent clusters and enable pathway engineering and to directly express metagenome gene clusters in heterologous hosts are providing the necessary tools to set up the basis for a new antibiotic discovery paradigm. Many recent excellent reviews cover each of the individual aspects of these new developments in the field and their impact on the identification of new antibiotics [19,20,21,22]. This short review is mostly focused on revisiting the most recent contributions in the field of actinomycetes in terms of their natural products, which have an impact on the discovery of novel scaffolds. These new approaches could bring new opportunities for the production of novel classes of compounds to address the current therapeutic needs in the multi-resistance era.

## 2. Exploiting Diversity of Cultured Actinomycetes

**Figure 1:**
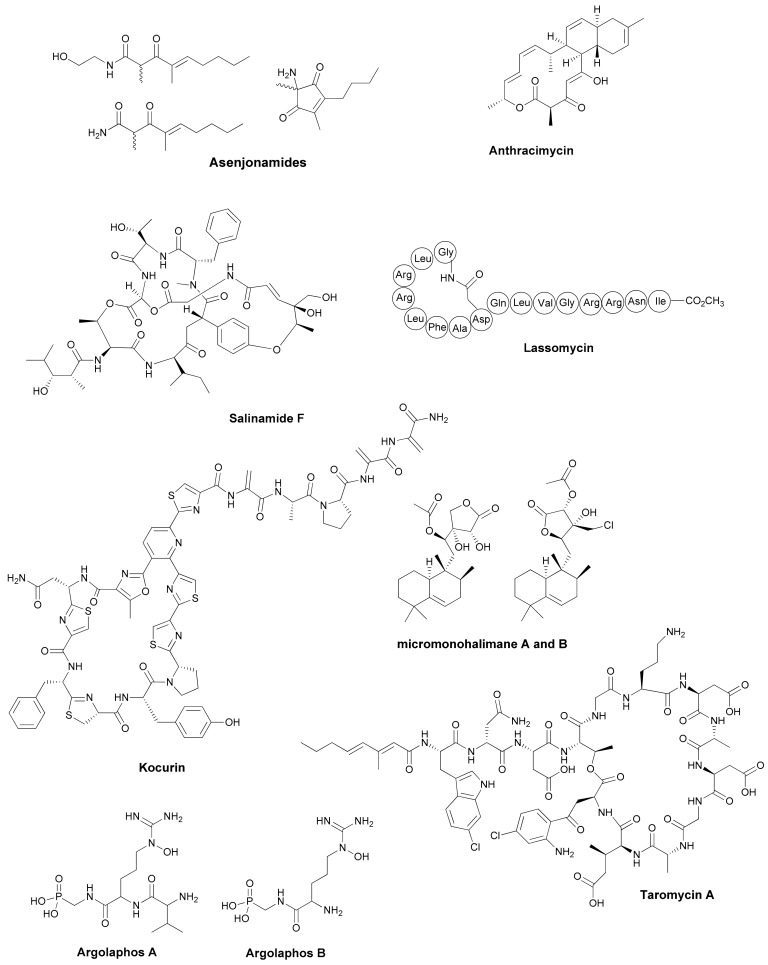

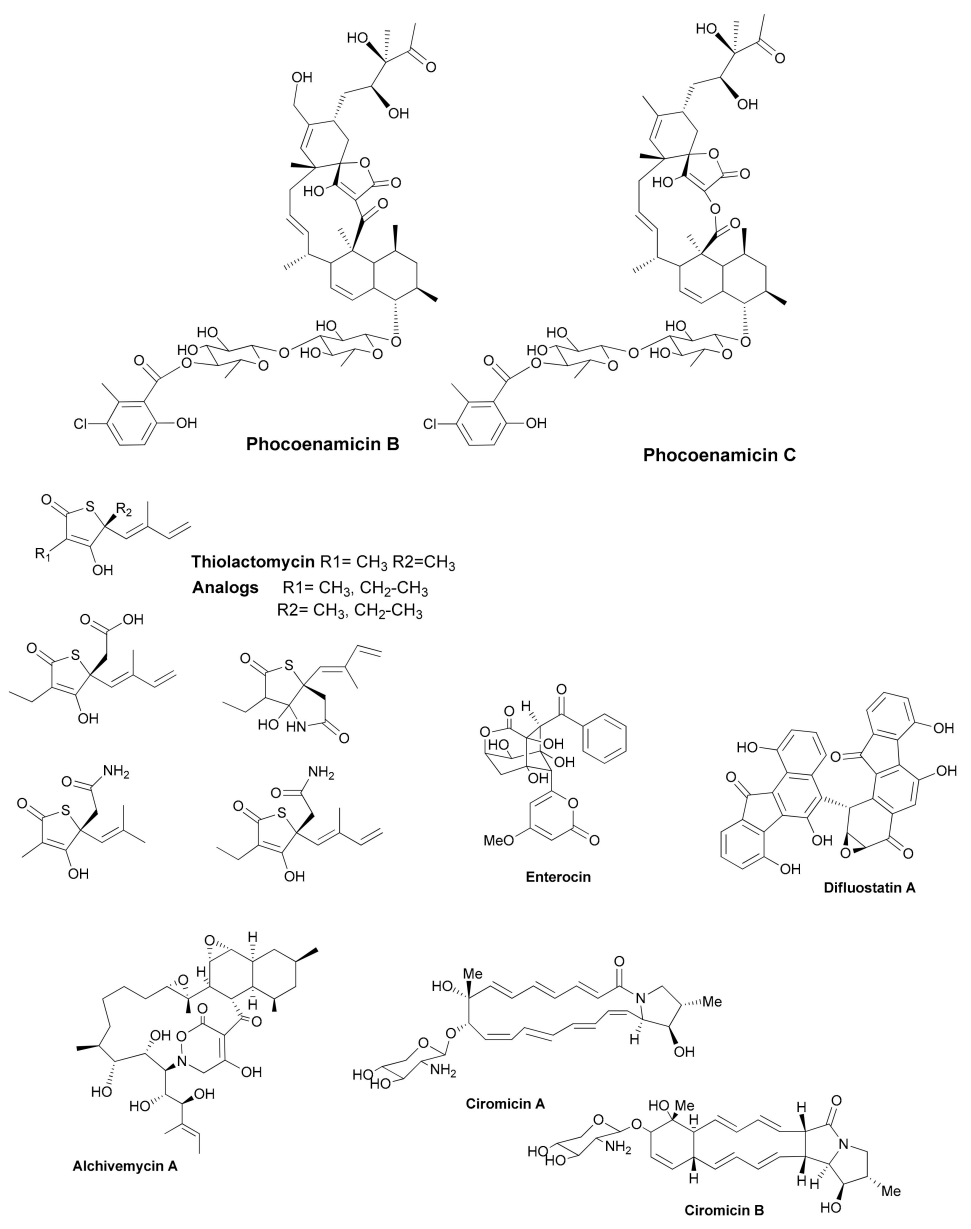
New antibiotics and analogs discovered from Actinomycetes.

The continued interest in exploring the environment to untap novel sources of microbial diversity has resulted in extensive prospective studies on a broad variety of sources, which ranges from specific terrestrial extreme environments to unique microbial assemblages in plant–host associations and marine ecosystems. The distribution of some microbial species presents biogeographic patterns that are mostly determined by micro-environmental conditions and the most recent efforts have been focused at exploiting these still poorly explored habitats to discover new chemical diversity. The exploration of the extreme conditions of desert and arid habitats, such as the Atacama desert with high salinity and high levels of UV radiation, has permitted the isolation of new species of actinomycetes that are well adapted to surviving under these conditions [23,24]. These actinomycetes communities have shown to produce a wide diversity of novel compounds from different natural product classes, such as the new ansamycins chaxamycins or the β-diketone polyketides asenjonamides A–C. This only names a few compounds with antibacterial activities that have been described recently [25,26] (Table 1) (Figure 1). Cave microbiomes are another pristine eco-system that have frequently been studied in the search for novel producing strains and many reports have highlighted the isolation of novel species of actinomycetes that produce bioactive compounds [27]. The actinobacterial diversity in calcite moonmilk deposits are one of the most recent examples showing the potential of these new isolates to create products with antibacterial activities [28]. Alternatively, the use of diffusion chamber methods to grow previously uncultured soil bacteria has permitted to isolate new antibiotics, such as lassomycin [29].

The marine environment has been traditionally another source of new actinomycetes [42,43]. The broad diversity of marine ecosystems, ranging from mangroves, shallow waters, deep sea sediments and associated invertebrates, have continued to attract the interest of microbiologists. Their isolation programs have ensured a continued discovery of new strains that produce new compounds or new analogs with biological activity (Table 1 and Figure 1) [30,31,32,33,34,43,44,45,46,47,48]. Despite this prolific description of marine-derived strains and new antibiotic producers, this environment remains poorly studied in terms of microbial diversity and functional diversity. Marine sediments have been the focus of recent studies, which has shown that actinobacteria are in fact only a minor component of this microbial community. More interestingly, these findings have suggested that the production of many of the secondary metabolites have a deep impact on the microbial community composition [49]. There is no doubt that the parallel advances in the metagenomic assessment of microbial diversity have allowed us to explore the dynamics of the microbial populations of interest in the communities currently being studied. These technologies are opening new avenues to investigate the potential roles of these members and the effects of the antibacterial compounds on the microbiome composition. The results derived from these studies will guide the isolation and selection of the most promising members of these microbial communities and their screening for the production of novel bioactive compounds.

## 3. Genomics Driven Discovery

**Figure 2:**
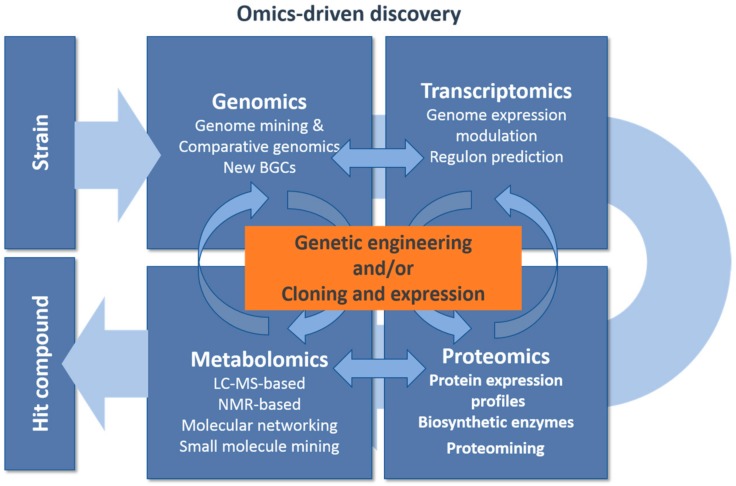
Omics-driven discovery.

In parallel, the exponential increase in the number of partial and complete genome sequencing projects on the actinomycetes species available in the public databases not only have confirmed their broad biosynthetic diversity across the different lineages, but also have enabled intensive genome mining approaches to untap new natural products scaffolds. The identification of new biosynthetic pathways of potentially interesting new molecules has fostered the search for new biosynthetic clusters (BGCs) on draft genomes, which has taken advantage of improved bioinformatic tools for cluster identification and gene annotation, such as AntiSMASH [50,51,52]. Furthermore, the increasing numbers of almost complete genomes has permitted the extensive comparative genomic analyses of members of this bacterial group, and the identification of the genomic components and the evolutionary history of many different species [53,54,55]. These comparative studies are revealing the existence of a core genome within some members of actinomycetes as well as a divergence of BGCs among the different lineages. These results are providing a basis for understanding the functional evolution of species as shown for *Streptomyces* [56,57]. Another relevant aspect of the impact of the increasing number of BGCs sequence information on antibiotic discovery is the possibility of developing specific targeted genome mining searches in genomic libraries based on specific genomic signatures related to the biosynthesis of privileged scaffolds or functionalizations that could drive the discovery of novel compounds and chemical spaces (Table 1 and Figure 1) [35,36]. The integration of genomics with transcriptomics, proteomics and metabolomics is providing unique information for assessing the functional evolution of actinomycetes species. These data are encouraging the development of new approaches for the expression and engineering of these biosynthetic pathways and the identification of new bioactive compounds from cultured actinomycetes not only from underexplored habitats but also from large microbial collections that are still the untapped treasures of biosynthetic diversity (Figure 2).

One of the major challenges that still remains in this context is the efficient cloning and expression of BGCs that are originally silent or poorly expressed in their natural host after the use of refactoring by the replacement of the regulatory elements and further detection of the synthetized compounds [37,58,59,60]. Thus far, many different in vitro DNA assembly or direct capture methods have been described to clone BGCs in heterologous hosts [38,39,61,62,63]. Many BGCs cannot be detected by the rule-based bioinformatic tools due to the absence of signature genes, but the application of prediction tools based on the frequencies of Pfam domains occurring in BGCs have improved the identification of additional clusters [51]. New genomic bacterial artificial chromosome (BAC) libraries built from large 100-Kb fragments of *Streptomyces* spp. genomic DNA are also being used in the high throughput functional screening approaches to identify non-predicted BGCs by heterologous expression [64].

## 4. Eliciting Production from Silent Pathways

The activation of silent BGCs in the well-characterized producing strains by direct gene manipulation is an alternative approach when the strains are amenable to genetic engineering. Many recent successful examples have been reported, which have described the use of the newest genetic engineering tools in modifying metabolic pathways, altering metabolic fluxes that are blocking unrelated metabolic pathways, inactivating transcriptional repressors, over-expressing pathway-specific activator genes or even multiplying the BGC copies in the original producing strains to increase titers [63,65,66,67]. The recent development of optimal ribosomal binding sequences and strong terminators to be applied in the control of the metabolic pathways of actinomycetes has opened new possibilities for gene expression modulation and represent promising tools for metabolic engineering (Table 2) [68].

Despite this success, a large proportion of wild-type and industrial strains remain recalcitrant to being manipulated and many silent BGCs cannot be directly activated using genetic tools. Culture-based approaches based on multiple nutritional conditions have been one of the most common methods employed to explore the media components required for the production of new compounds. The concept of using small molecules as elicitors by perturbing biological systems and signaling pathways dates back several decades and has been extensively used to activate silent or poorly expressed pathways with a broad range of small molecules, including the sub-inhibitory concentrations of antibiotics [69,70]. The first large scale elicitor screening reported was performed with more than 30,000 compounds on *S. coelicolor* and it identified a small number of compounds that are able to stimulate the production of some of the secondary metabolites by several times [89]. Many examples of hormesis have been described with sub-inhibitory concentrations of antibiotics and other well-known bioactive natural products, which has elicited a response that is associated with the major activation of secondary metabolism, induction of cryptic gene clusters and production of novel compounds [70,90]. The activation effect cannot be predicted from the antibiotic mode of actions and the lack of universal effectors to awaken all silent BGCs has added another level of complexity in the identification of new effector molecules [70,71]. The same effect is pursued by co-cultivation, which is an approach that has been used extensively with many different types of cultivation formats and strain combinations. The approach takes advantage of the effect that small concentrations of signaling or antibiotic molecules from one of the strains can have on another strain. The difficulty to scale-up this approach as a general method is related to the impossibility of predicting which combinations will result in an effective response, which normally does not account for more than 15–20% of the cases studied [71]. Mycolic acids have been shown to play a role in the physical interaction and the activation of some silent pathways. New antibacterial compounds, such as alchivemycin A and B, arcyriaflavin E or ciromicins, were described after co-culturing different *Rhodoccoci* with the species of *Streptomyces*, *Tsukamurella* and *Nocardiopsis* (Table 1) (Figure 1) [40,41]. In other situations, the induction does not require cell-to-cell interaction and is only mediated by diffusible small effector molecules. One of the most recent examples is the production of the cryptic natural product keyicin from the co-cultivation of the producer *Micromonospora* strain with *Rhodococcus* [91] (Table 2).

Most recent studies on the differential metabolomic analysis of metabolites in response to cultivation conditions have shown that the chemical potential of actinomycetes is far from being fully characterized [92]. From a methodological perspective, the modern LC-MS and NMR analytical tools and differential metabolomic analysis have been the determinant for detecting the production of novel compounds in complex mixtures and mapping the response to external chemical signals. Comparative metabolomics have been efficiently used to identify the induced expression of secondary metabolites from *S. coelicolor* cryptic genes resulting from the exposure to multiplexed perturbations and to identify the subsets of primary and secondary metabolites that respond similarly across a large variety of stimuli [72]. The major challenge for these methods is related to the identification of novel bioactive molecules within the complexity of the metabolomic profiles. New dereplication and identification approaches are continuously being developed, which are based on the similarity of MS/MS patterns in natural product databases and NMR-based metabolomics [73,74,75]. Proteomining is another method developed to support this identification. The analysis links natural products to biosynthetic enzymes as it correlates protein expression profiles of biosynthetic enzymes to the metabolome of the producing strains based on the statistical analysis of strains cultured under different conditions [76,77].

## 5. Harnessing Regulation of Primary and Secondary Metabolisms

The production of secondary metabolites in actinomycetes is tightly regulated and responds to external stimuli from the environment. This regulation is the result of different classes of pathway-specific regulatory elements involving two-component systems, extra-cytoplasmic sigma factors or pathway specific regulators, such as the most recently described LmbU family [78,79,80]. Triggering the expression of BGCs frequently involves an additional transcriptional response through the master regulators involved in global regulatory metabolic networks that are not always pathway specific [81]. Understanding the right combinations of regulatory elements and transcription factors that regulate a BGC has been proposed as the “cracking the code” approach to be followed in identifying the key regulatory signals and the eliciting signals needed to set up culture conditions to activate a specific BGC [83]. The regulon predictor program PREDetector was developed to identify signaling cascades deduced from the in silico searches of regulatory elements, which revealed that primary metabolism transcription factors were also involved in controlling pathway-specific regulators [84]. One of the best examples are the pleiotropic regulators, DasR and CebR, that control the uptake of chitin and cellulose, for which responsive elements were identified upstream of many pathway-specific regulators [85,86,87]. The increasing number of available new genomes are enabling the new in silico approaches derived from comparative genomics to detect specific binding motifs of transcription factor orthologues [82]. Regulon prediction has been proposed as a successful strategy to identify the regulatory networks involved in the control of BGC expression and the most promising strains to be explored for the induction of silent pathways. This finely tuned participation of regulatory requirements for the specialized metabolite production also requires the precise provision of chemical precursors from primary metabolism. Recent reports highlight the role of primary metabolism gene expansions in secondary metabolite producing strains with impact on metabolic adaptation and strain fitness and how they may represent another target for future genetic engineering interventions to improve production and activate silent pathways [88] (Table 2).

## 6. Conclusions and Future Prospects

Microbial prospection studies have continued to reveal that there is a huge and still poorly explored diversity of actinomycetes in the environment that is waiting to be mined for the isolation of new bioactive compounds. In addition, thousands of selected strains that are preserved in microbial collections and distributed across laboratories worldwide also should be revisited as they represent a unique reservoir of silent biosynthetic diversity that traditional approaches did not manage to unlock in its full extension. The continued increase in new genome sequences and the development of new genome-mining and genome-directed engineering tools to trigger the production of new natural products are paralleling the development of integrated analytical tools and open access databases. The application of comparative genomics, metabolomics and proteomics to culture-based studies is providing a wealth of knowledge on the physiology and the regulation systems, opening new avenues to approach the activation of silent or poorly expressed pathways. There is no doubt that all these advances are setting the new foundations for a new paradigm in natural product discovery, especially from actinomycetes. A sustained and integrated multidisciplinary research effort should respond to the major challenge of discovering new chemical classes of antibiotics that will be required to replenish the preclinical development pipeline in the near future.

## Figures and Tables

**Table 1:** New antibiotics and analogs described from Actinomycetes since 2013, following different mining approaches.

Antibiotic	Structural Class	Producing Species	Antibiotic Spectrum	Discovery Approach	Reference
Asenjonamides A–C	di-ketone polyketides	*S. asenjonii*	Gram positive/ negative	Extreme environment	[26]
Lassomycin	cyclic peptide	*Lentzea kentukyensis*	*M. tuberculosis*	Diffusion chambers	[29]
Anthracimycin	tricarboxilic	*Streptomyces* sp.	Gram positive	Marine source	[30]
Salinamide F	depsipeptide	*Streptomyces* sp.	Gram positive/ negative	Marine source	[**31**]
Kocurin	thiazolylpeptide	*Kocuria lacustris* /*Micrococcus* sp.	Gram positive	Marine source	[32]
Micromonohalimanes A and B	diterpenoids	*Micromonospora* sp.	Gram positive	Marine source	[33]
Phocoenamicins B and C	spirotetronates	*Micromonospora* sp.	Gram positive	Marine source	[34]
Argolaphos A/B	phosphonopeptide	*Streptomyces monomycini*	Gram positive/negative	Genome-driven	[35]
Thiolactomycin and analogs	thiotetronic acids	*Salinispora/S. afghaniensis*	Gram positive	Genome-driven	[36]
taromycin A	lipopetide	*Saccharomonospora* sp.	Gram positive	Genome-driven	[37]
Enterocin	polyketide	*Salinispora pacifica*	Gram positive	Genome-driven	[38]
Difluostatin A	angucycline	*Micromonospora rosaria*	Gram positive	Genome-driven	[39]
Alchivemycin A and B	heterocyclic	*S. endus + Tsukamurella pulmonis*	Gram positive	Co-cultivation	[40]
Ciromicins	polyketide	*Nocardiopsis* sp. *+ Rhodococcus wratislaviensis*	Not determined	Co-cultivation	[41]

**Table 2:** Strategies to elicit antibiotic production in actinomycetes.

Eliciting Production Approaches	Methods and Targets	References
Genetic engineering:	Genome expression modulation: Transcriptional repressors inactivation; Transcriptional activators overexpression Optimized ribosomal binding sequences Strong terminators Increase BGCs copies Alter metabolic fluxes	[63,65,66,67,68]
Culture-based approaches:	Small molecule signaling: Sub-inhibitory small molecule elicitors Co-cultivation: cell–cell signaling	[69,70][71]
Analytical mining:	Comparative metabolomics: LC-MS-based metabolomics NMR-based metabolomics Proteomining	[72,73,74,75][75][76,77]
Regulation of primary and secondary metabolism	Pathway specific regulatory elements: Two-component systems Sigma factors Pathways specific regulators	[78,79,80]
	Global regulatory metabolic networks Master regulators	[81]
	Comparative genomics: Identification of Transcription factor orthologs	[82]
	Regulon prediction: identification of regulatory networks Primary metabolism transcription factors Signaling cascades Pleiotropic regulators	[83,84][85,86,87]
	Primary metabolism gene expansion	[88]
